# Patterns of staging, treatment, and mortality in gastric, colorectal, and lung cancer among older adults with and without preexisting dementia: a Japanese multicentre cohort study

**DOI:** 10.1186/s12885-022-10411-y

**Published:** 2023-01-19

**Authors:** Toshitaka Morishima, Yoshihiro Kuwabara, Mari Kajiwara Saito, Satomi Odani, Haruka Kudo, Mizuki Kato, Kayo Nakata, Isao Miyashiro

**Affiliations:** Cancer Control Center, Osaka International Cancer Institute, 3-1-69 Otemae, Chuo-ku, 541-8567 Osaka, Japan

**Keywords:** Administrative claims data, Alzheimer disease, Dementia, Geriatrics, Multicenter study, Neoplasms, Practice patterns, Registries, Treatment outcome

## Abstract

**Background:**

Little is known about dementia’s impact on patterns of diagnosis, treatment, and outcomes in cancer patients. This study aimed to elucidate the differences in cancer staging, treatment, and mortality in older cancer patients with and without preexisting dementia.

**Methods:**

Using cancer registry data and administrative data from 30 hospitals in Japan, this multicentre retrospective cohort study examined patients aged 65–99 years who were newly diagnosed with gastric, colorectal, or lung cancer in 2014–2015. Dementia status (none, mild, and moderate-to-severe) at the time of cancer diagnosis was extracted from clinical summaries in administrative data, and set as the exposure of interest. We constructed multivariable logistic regression models to analyse cancer staging and treatment, and multivariable Cox regression models to analyse three-year survival.

**Results:**

Among gastric (*n* = 6016), colorectal (*n* = 7257), and lung (*n* = 4502) cancer patients, 5.1%, 5.8%, and 6.4% had dementia, respectively. Patients with dementia were more likely to receive unstaged and advanced-stage cancer diagnoses; less likely to undergo tumour resection for stage I, II, and III gastric cancer and for stage I and II lung cancer; less likely to receive pharmacotherapy for stage III and IV lung cancer; more likely to undergo tumour resection for all-stage colorectal cancer; and more likely to die within three years of cancer diagnosis. The effects of moderate-to-severe dementia were greater than those of mild dementia, with the exception of tumour resection for colorectal cancer.

**Conclusion:**

Older cancer patients with preexisting dementia are less likely to receive standard cancer treatment and more likely to experience poorer outcomes. Clinicians should be aware of these risks, and would benefit from standardised guidelines to aid their decision-making in diagnosing and treating these patients.

**Supplementary Information:**

The online version contains supplementary material available at 10.1186/s12885-022-10411-y.

## Background

Population ageing is increasing worldwide, and at least 30% of the global population will be aged 65 years or older by 2050 [1]. In particular, Japan is currently the world’s most ‘super-aged’ society with 28.4% of its population aged 65 years or older in 2020 [2]. An ageing population poses a variety of unique challenges for health care services, such as a rising prevalence of dementia and other cognitive disorders. The worldwide prevalence of dementia is projected to increase from 46.8 million people in 2015 to 74.7 million people in 2030 [3]. Dementia encompasses a group of conditions that progressively impair cognitive function (e.g., memory, communication, and decision-making abilities), and its aetiological forms include Alzheimer’s disease, vascular dementia, Lewy body dementia, and frontotemporal dementia [4]. In addition, ageing is also a risk factor for the development of cancer. More than 28 million people globally are expected to have cancer in 2040, which represents a 47% increase from the 19.3 million people in 2020 [5]. Consequently, the number of older adults living with both dementia and cancer is expected to rise [6].

While the concomitant occurrences of these two conditions are well documented, less is known about the complexities of cancer pathways from diagnosis to death in newly diagnosed cancer patients with preexisting dementia [7–9]. According to the few available studies, cancer patients with preexisting dementia are more likely to be diagnosed at an unknown or later stage of cancer [10–12] and less likely to receive cancer treatment [11–17] when compared with their non-dementia counterparts. Due to factors such as poorer cancer management, patients with dementia have shorter survival times than those without dementia [10, 11, 13, 15–18]. Among the studies on oncological patterns in cancer patients with preexisting dementia, none have considered the severity of dementia using multicentre large-scale samples. This was because most of those studies identified dementia based on diagnosis codes, prescribed medication, or a combination of both as recorded in health insurance claims data [10–15, 18]. Thus, the role of preexisting dementia as a comorbidity factor in cancer patients remains unclear, and further investigations are warranted to assess its severity-dependent impact on cancer pathways in this growing ‘dual-condition’ population [9].

As dementia is more prevalent in older adults, discerning the extent to which it adversely affects cancer pathways has implications for both oncological and geriatric care. Furthermore, the insights from real-world settings could have clinical and research applications for health care services. The aim of this study was to elucidate the differences in cancer pathways from diagnosis to death between older adults with and without preexisting dementia. Specifically, we sought to characterise the differences in cancer stage, treatment, and subsequent all-cause mortality in patients newly diagnosed with gastric, colorectal, or lung cancer.

## Methods

### Study design and data source

We conducted a multicentre retrospective cohort study of cancer patients residing in Osaka Prefecture, Japan. The study was performed using a database comprising hospital-based cancer registry data, administrative data, and population-based cancer registry data. First, clinical data from a hospital-based cancer registry were linked with administrative data produced by hospitals for reimbursements under Japan’s Diagnosis Procedure Combination/Per-Diem Payment System. The details of this record-linked database have been reported previously [19–26]. Briefly, the hospital-based cancer registry collects patient demographic information, as well as information on the diagnosis and treatment of newly diagnosed cancer cases; this includes the date of diagnosis, topographical and morphological codes based on the International Classification of Diseases for Oncology Third Edition (ICD-O-3), cancer stage at the time of diagnosis based on the Seventh Edition of the Union for International Cancer Control staging system, and cancer treatment modality. Cancer treatment refers to the initially planned course of treatment that occurred within four months of cancer diagnosis. The administrative data contained inpatient clinical summaries for hospitalisation episodes. In addition to the hospital-based cancer registry data and administrative data, our study database also incorporated data from the Osaka Cancer Registry, a population-based cancer registry that collects information on vital statuses of Osaka Prefecture residents using death certificates and official resident registrations. Using these data, we identified residents diagnosed with cancer in 2014 and 2015; their vital statuses were tracked in May 2018 and May 2019, respectively.

The study database, which was organisationally supported by the Council for Coordination of Accredited Cancer Hospitals, comprised data that were voluntarily provided by 31 hospitals in Osaka Prefecture. These hospitals are accredited as cancer hospitals by the national or prefectural government, and treat approximately half of all newly diagnosed cancer patients residing in the study region.

### Study population

Using the hospital-based cancer registry data, we first identified 18,018 eligible subjects who (a) were diagnosed with gastric (ICD-O-3 topographical codes: C16.x), colorectal (C18.x–C20.x), or lung cancer (C33.x–C34.x) between April 1, 2014 and December 31, 2015; (b) were aged 65–99 years at the time of cancer diagnosis; (c) received cancer treatment at any of the 31 study hospitals; and (d) had at least one cancer-specific hospitalisation episode within 90 days before or after a cancer diagnosis as identified in the administrative data. The three cancer sites were chosen due to their relatively high prevalence in older adults residing in the study region. When a single patient had two or more cancer records for one site, we selected his/her earliest record of the most advanced-stage cancer. Cancer treatment included best supportive care as well as treatment with curative intent. We excluded tumours of the three sites if they were diagnosed as sarcoma (ICD-O-3 morphological codes: 8800–9044, 9120–9262, or 9540–9581; *n* = 147), haematological tumour (9590–9989; *n* = 91), or melanoma (8720–8790; *n* = 3). In addition, patients were excluded if they had missing data for dementia status at the time of cancer diagnosis (*n* = 2). One of the study hospitals had no eligible patients, and the final study population comprised 17,775 patients from 30 hospitals.

### Preexisting dementia

Dementia status at the time of cancer diagnosis was analysed as the exposure of interest. Japanese hospitals are required to include dementia status in clinical summaries for inpatients aged 65 years or older discharged on or after April 1, 2014. Dementia status is evaluated upon hospital admission using a scale described below. For each patient identified in the hospital-based cancer registry data, we searched the clinical summaries in the administrative data for the cancer-specific hospitalisation episode closest to the cancer diagnosis (designated the index hospitalisation) to determine dementia status.

Dementia status was evaluated using a dementia scale that assigns ranks to persons based on their symptoms and degree of independence in activities of daily living: no dementia (rank 0); having symptoms of dementia, but can live independently in one’s home and community without assistance (rank I); having symptoms of dementia, but can live independently in one’s home and community with assistance (rank II); having symptoms of dementia that sometimes affect daily life such that occasional caregiving is required (rank III); having symptoms of dementia that frequently affect daily life such that full-time caregiving is required (rank IV); and having severe symptoms of dementia that require specialised medical treatments (rank M) [27]. This scale has been shown to have good reliability and validity [27]. The ranks are aggregated into three categories (rank 0, ranks I to II, ranks III to IV or M); for this study, we considered these categories to indicate the severity of dementia (no dementia, mild dementia, and moderate-to-severe dementia, respectively).

### Cancer staging

Cancer stage at the time of diagnosis was determined using pathological staging. However, clinical staging was used for patients who did not undergo surgical resection for their cancer or had received neoadjuvant therapy prior to surgical resection. Patients with missing information on cancer stage in the hospital-based cancer registry data were classified as having ‘unstaged’ cancer. To assess if there were differences in staging between patients with and without dementia, we performed multivariable logistic regression analyses where the outcome was the receipt of an unstaged cancer diagnosis (vs. staged cancer). Dementia status was examined as the explanatory variable of interest, and the covariates included age (65–69, 70–74, 75–79, 80–84, ≥ 85 years), sex, and comorbidities at the time of diagnosis. Information on age and sex were obtained from the hospital-based cancer registry data, and information on comorbidities was acquired from the clinical summaries in the administrative data corresponding to the index hospitalisation. Comorbidities were measured using updated Charlson Comorbidity Index (CCI) scores based on International Classification of Diseases, Tenth Revision codes [28, 29]. These scores were calculated as the sum of the individual component scores (ranging from 1 to 4) for 10 major diseases (e.g., heart failure and renal disease) associated with increased mortality. Dementia was excluded from CCI scoring. In addition, metastatic cancer was also excluded from CCI scoring because it may be associated with cancer stage in the target cancers. Patients were categorised as follows: no comorbidity (CCI score: 0), moderate comorbidities (1–2), and severe comorbidities (≥ 3). The measurement method is described in further detail in our previous study [19].

Next, patients with unstaged cancer (*n* = 233) were excluded, and cancer stages were categorised into early stage (0 and I) and advanced stage (II, III, and IV). We performed multivariable logistic regression analyses where the outcome was the receipt of an advanced-stage cancer diagnosis (vs. early-stage cancer). Dementia status was examined as the explanatory variable of interest, and the covariates included age, sex, and comorbidities.

### Cancer treatment

We examined cancer treatment after excluding patients with unstaged cancer (*n* = 233), patients who were diagnosed with small cell lung cancer (ICD-O-3 morphological codes: 8041–8045; *n* = 461), and patients who were diagnosed with stage 0 lung cancer (*n* = 8). These patients were excluded because standard treatments could not be determined according to cancer stage, because of major differences with non-small cell lung cancer (NSCLC) patients, and because of the small sample size, respectively. Cancer treatment included endoscopic resection, open surgical resection, laparoscopic resection, thoracoscopic resection, pharmacotherapy, and radiotherapy; information on these treatments was obtained from the hospital-based cancer registry data. The first four modalities were collectively categorised as ‘tumour resection’. Debulking surgery and radical resection were also included in tumour resection. On the other hand, palliative procedures that relieved symptoms but did not reduce tumour mass (e.g., bypass surgery and endoscopic stent placement) were not included in cancer treatment.

First, we performed multivariable logistic regression analyses where the outcome variable was the receipt of any cancer treatment modality (vs. no treatment) included in the initially planned course [14]. Next, we performed multivariable logistic regression analyses where the outcome variable was the receipt of a standard treatment modality (vs. no standard treatment). To identify the standard treatment modalities (tumour resection, pharmacotherapy, and radiotherapy) for each cancer site and stage among older patients in current real-world settings, we performed preliminary analyses to investigate the most common treatment modality for each stage of cancer using our dataset. We found that tumour resection was most common for stage I (91%), II (53%), and III (32%) gastric cancer; pharmacotherapy was most common for stage IV (42%) gastric cancer; tumour resection was most common for stage 0 (99%), I (85%), II (84%), III (42%), and IV (27%) colorectal cancer; tumour resection was most common for stage I (74%) and II (48%) NSCLC; and pharmacotherapy was most common for stage III (23%) and IV (46%) NSCLC. Therefore, tumour resection was regarded as the standard treatment modality for stage I, II, and III gastric cancer, all stages of colorectal cancer, and stage I and II NSCLC. Pharmacotherapy was regarded as the standard treatment modality for stage IV gastric cancer and stage III and IV NSCLC. In these multivariable logistic regression models, dementia status was examined as the explanatory variable of interest, and the covariates included age, sex, comorbidities, and stage. In addition, we constructed stage-stratified multivariable logistic regression models where the outcome was the receipt of a standard treatment modality (vs. no standard treatment) for each stage. Dementia status was examined as the explanatory variable of interest, and the covariates included age, sex, and comorbidities.

### Mortality

We constructed Cox proportional hazards regression models where the outcome of interest was overall survival time for a maximum follow-up period of three years. The duration of follow-up was defined as the period between the date of cancer diagnosis and the date of death from any cause. Patients were censored at the date of the last follow-up with an ‘alive’ status from the registry data or administrative data, whichever was later. Dementia status was examined as the explanatory variable of interest, and the covariates included age, sex, comorbidities, stage, and histology. ‘Unstaged’ was included in cancer stage because we considered it to have potential prognostic value. Histology was only included as a covariate for lung cancer, and was classified into small cell lung cancer (ICD-O-3 morphological codes: 8041–8045) and NSCLC (all other codes). Patients with no follow-up for vital status (*n* = 7) and patients with stage 0 lung cancer (*n* = 8) were excluded from the survival analyses.

### Other statistical procedures

Crude percentages were used to compare the distribution of demographic and tumour characteristics among the three dementia status groups (no dementia, mild dementia, and moderate-to-severe dementia). Adjusted odds ratios and adjusted hazard ratios were reported with their 95% confidence intervals for the logistic regression models and Cox proportional hazards regression models, respectively. All analyses were performed separately for each of the three cancer sites. All *P* values were two-sided, and *P* < 0.05 was considered statistically significant. Survival analyses were performed using SAS 9.4 (SAS Institute Inc., Cary, NC, USA), and all other analyses were performed using IBM SPSS Statistics version 22 (IBM Corp., Armonk, NY, USA).

## Results

The study population comprised 6016 gastric cancer patients, 7257 colorectal cancer patients, and 4502 lung cancer patients. The patients’ demographic characteristics are presented according to cancer site and dementia status in Table 1. There were 224 (3.7%), 324 (4.5%), and 216 (4.8%) patients with mild dementia in the gastric cancer, colorectal cancer, and lung cancer groups, respectively. Next, there were 80 (1.3%), 98 (1.4%), and 73 (1.6%) patients with moderate-to-severe dementia in the gastric cancer, colorectal cancer, and lung cancer groups, respectively. In all three cancer sites, a higher proportion of patients without dementia were younger, male, and had no comorbidities when compared with patients with dementia.

**Table 1:** Demographic and tumour characteristics of older adults according to cancer site and dementia status

	Gastric cancer (*n* = 6016)			Colorectal cancer (*n* = 7257)			Lung cancer (*n* = 4502)		
	No dementia	Mild dementia	Moderate-to-severe dementia	No dementia	Mild dementia	Moderate-to-severe dementia	No dementia	Mild dementia	Moderate-to-severe dementia
Total	5712 (100)	224 (100)	80 (100)	6835 (100)	324 (100)	98 (100)	4213 (100)	216 (100)	73 (100)
Age, years									
65–69	1374 (24.1)	7 (3.1)	3 (3.8)	1796 (26.3)	11 (3.4)	5 (5.1)	1059 (25.1)	16 (7.4)	3 (4.1)
70–74	1602 (28.0)	20 (8.9)	6 (7.5)	1921 (28.1)	39 (12.0)	6 (6.1)	1317 (31.3)	32 (14.8)	6 (8.2)
75–79	1477 (25.9)	58 (25.9)	11 (13.8)	1666 (24.4)	70 (21.6)	24 (24.5)	1011 (24.0)	52 (24.1)	18 (24.7)
80–84	865 (15.1)	77 (34.4)	23 (28.7)	984 (14.4)	107 (33.0)	28 (28.6)	600 (14.2)	62 (28.7)	15 (20.5)
≥ 85	394 (6.9)	62 (27.7)	37 (46.3)	468 (6.8)	97 (29.9)	35 (35.7)	226 (5.4)	54 (25.0)	31 (42.5)
Sex									
Male	4046 (70.8)	135 (60.3)	48 (60.0)	4044 (59.2)	175 (54.0)	38 (38.8)	2879 (68.3)	147 (68.1)	47 (64.4)
Comorbidity									
No comorbidity	4338 (75.9)	147 (65.6)	63 (78.8)	5493 (80.4)	238 (73.5)	75 (76.5)	2883 (68.4)	139 (64.4)	48 (65.8)
Moderate comorbidities	1197 (21.0)	68 (30.4)	15 (18.8)	1190 (17.4)	69 (21.3)	22 (22.4)	1155 (27.4)	66 (30.6)	22 (30.1)
Severe comorbidities	177 (3.1)	9 (4.0)	2 (2.5)	152 (2.2)	17 (5.2)	1 (1.0)	175 (4.2)	11 (5.1)	3 (4.1)
Cancer stage									
0	—	—	—	1853 (27.1)	55 (17.0)	8 (8.2)	8 (0.2)	0 (0.0)	0 (0.0)
I	3608 (63.2)	115 (51.3)	33 (41.3)	1431 (20.9)	54 (16.7)	10 (10.2)	1397 (33.2)	53 (24.5)	7 (9.6)
II	535 (9.4)	32 (14.3)	6 (7.5)	1326 (19.4)	75 (23.1)	30 (30.6)	392 (9.3)	11 (5.1)	3 (4.1)
III	583 (10.2)	30 (13.4)	9 (11.3)	1339 (19.6)	71 (21.9)	20 (20.4)	791 (18.8)	30 (13.9)	13 (17.8)
IV	908 (15.9)	40 (17.9)	21 (26.3)	843 (12.3)	57 (17.6)	24 (24.5)	1571 (37.3)	111 (51.4)	39 (53.4)
Unstaged	78 (1.4)	7 (3.1)	11 (13.8)	43 (0.6)	12 (3.7)	6 (6.1)	54 (1.3)	11 (5.1)	11 (15.1)
Small cell lung cancer									
Yes	—	—	—	—	—	—	435 (10.3)	21 (9.7)	5 (6.8)
Tumour resection^a^									
Yes	3868 (67.7)	146 (65.2)	26 (32.5)	4908 (71.8)	253 (78.1)	65 (66.3)	1354 (32.1)	33 (15.3)	5 (6.8)
Pharmacotherapy									
Yes	466 (8.2)	13 (5.8)	3 (3.8)	136 (2.0)	7 (2.2)	1 (1.0)	1091 (25.9)	37 (17.1)	9 (12.3)
Radiotherapy									
Yes	6 (0.1)	0 (0.0)	0 (0.0)	12 (0.2)	1 (0.3)	1 (1.0)	276 (6.6)	29 (13.4)	5 (6.8)
All-cause mortality within 3 years of cancer diagnosis									
Deaths	1773 (31.0)	133 (59.4)	67 (83.8)	1412 (20.7)	156 (48.1)	74 (75.5)	2371 (56.3)	184 (85.2)	71 (97.3)
Median follow-up period, years (IQR)	3.00 (1.99–3.00)	1.96 (0.37–3.00)	0.47 (0.16–1.94)	3.00 (3.00–3.00)	3.00 (0.89–3.00)	1.19 (0.25–2.88)	2.14 (0.69–3.00)	0.58 (0.16–1.78)	0.22 (0.08–0.58)

Values are n (%) unless stated otherwise

*IQR *interquartile range

^a^Tumour resection includes endoscopic resection, open surgical resection, laparoscopic resection, and thoracoscopic resection

The tumour characteristics are also provided in Table 1. In all three cancer sites, a higher proportion of patients without dementia were diagnosed with stage 0 or I cancer, whereas a lower proportion of patients without dementia were diagnosed with stage IV or unstaged cancer when compared with patients with dementia. Among the gastric cancer and lung cancer patients, a higher proportion of patients without dementia underwent tumour resection and pharmacotherapy. Overall, there were fewer deaths in patients without dementia during the three-year follow-up period than patients with dementia.

### Impact of dementia on cancer staging

The associations between dementia status and cancer staging at the time of diagnosis are summarised in Table 2. Model 1 evaluated the associations between dementia status and receiving an unstaged cancer diagnosis after adjusting for age, sex, and comorbidities. Patients with moderate-to-severe dementia had significantly higher odds of being diagnosed with unstaged gastric cancer than those without dementia. Patients with mild or moderate-to-severe dementia had significantly higher odds of being diagnosed with unstaged colorectal cancer or lung cancer than those without dementia. The effect size was greater for moderate-to-severe dementia than mild dementia, as shown in the higher point estimates of the adjusted odds ratios. Model 2 evaluated the associations between dementia status and receiving an advanced-stage cancer diagnosis (excluding patients with unstaged cancer) after adjusting for age, sex, and comorbidities. For all three cancer sites, patients with mild or moderate-to-severe dementia had significantly higher odds of being diagnosed with advanced-stage cancer than those without dementia. The effect size was greater for moderate-to-severe dementia than mild dementia, and varied among the cancer sites.

**Table 2:** Associations between dementia status and diagnoses of unstaged or advanced-stage cancer in older adults

	Model 1: Adjusted odds ratios of being diagnosed with unstaged cancer						
	**Gastric cancer** (*n* = 6016)		**Colorectal cancer** (*n* = 7257)		**Lung cancer** (*n* = 4502)		
	**Odds ratio** **(95% CI)**	*P* value	**Odds ratio** **(95% CI)**	*P* value	**Odds ratio** **(95% CI)**	*P* value	
No dementia	Reference		Reference		Reference		
Mild dementia	1.38 (0.61–3.10)	0.442	3.63 (1.81–7.27)	< 0.001	2.62 (1.30–5.27)	0.007	
Moderate-to-severe dementia	5.27 (2.55–10.91)	< 0.001	6.35 (2.52–16.05)	< 0.001	7.47 (3.49–16.01)	< 0.001	
	Model 2: Adjusted odds ratios of being diagnosed with advanced-stage cancer^a^						
	**Gastric cancer** (*n* = 5920)		**Colorectal cancer** (*n* = 7196)		**Lung cancer** (*n* = 4426)		
	**Odds ratio** **(95% CI)**	*P* value	**Odds ratio** **(95% CI)**	*P* value	**Odds ratio** **(95% CI)**	*P* value	
No dementia	Reference		Reference		Reference		
Mild dementia	1.49 (1.13–1.97)	0.005	1.47 (1.15–1.87)	0.002	1.40 (1.01–1.95)	0.043	
Moderate-to-severe dementia	1.79 (1.10–2.89)	0.019	3.14 (1.85–5.30)	< 0.001	3.78 (1.70–8.40)	0.001	

All odds ratios are adjusted for age, sex, and comorbidities. Model 2 only included patients who received cancer staging

*CI* confidence interval

^a^Advanced stage includes stages II, III, and IV

### Impact of dementia on cancer treatment

Table 3 presents the associations between dementia status and cancer treatment after adjusting for age, sex, comorbidities, and stage. Model 3 evaluated the associations between dementia status and any cancer treatment, irrespective of modality. Patients with moderate-to-severe dementia had significantly lower odds of receiving any cancer treatment for gastric cancer than those without dementia. In contrast, patients with mild dementia had significantly higher odds of receiving any cancer treatment for colorectal cancer than those without dementia. Patients with mild or moderate-to-severe dementia had significantly lower odds of receiving any cancer treatment for NSCLC. Model 4 evaluated the associations between dementia status and tumour resection. Patients with moderate-to-severe dementia had significantly lower odds of undergoing tumour resection for stage I, II, and III gastric cancer than those without dementia. Patients with mild dementia had significantly higher odds of undergoing tumour resection for stage 0, I, II, III, and IV colorectal cancer than those without dementia. Patients with mild or moderate-to-severe dementia had significantly lower odds of undergoing tumour resection for stage I and II NSCLC than those without dementia. No significant association was found between mild dementia and tumour resection for stage I, II, and III gastric cancer or between moderate-to-severe dementia and tumour resection for colorectal cancer. In Model 5, we examined the association between dementia status and pharmacotherapy. Patients with mild or moderate-to-severe dementia had significantly lower odds of receiving pharmacotherapy for stage III and IV NSCLC than those without dementia. No significant association was found between dementia status and pharmacotherapy for stage IV gastric cancer. The effect size was greater for moderate-to-severe dementia than mild dementia, with the exception of tumour resection for colorectal cancer.

**Table 3:** Associations between dementia status and cancer treatment in older adults

	Model 3: Adjusted odds ratios of receiving any modality of cancer treatment^a^					
	**Stage I, II, III, and IV gastric cancer** (*n* = 5920)		**Stage 0, I, II, III, and IV colorectal cancer** (*n* = 7196)		**Stage I, II, III, and IV NSCLC** (*n* = 3966)	
	**Odds ratio** **(95% CI)**	*P* value	**Odds ratio** **(95% CI)**	*P* value	**Odds ratio** **(95% CI)**	*P* value
No dementia	Reference		Reference			
Mild dementia	0.86 (0.60–1.22)	0.386	1.56 (1.11–2.19)	0.011	0.49 (0.36–0.68)	< 0.001
Moderate-to-severe dementia	0.17 (0.09–0.30)	< 0.001	0.90 (0.53–1.52)	0.689	0.24 (0.13–0.45)	< 0.001
	Model 4: Adjusted odds ratios of undergoing tumour resection^b^					
	**Stage I, II, and III gastric cancer** (*n* = 4951)		**Stage 0, I, II, III, and IV colorectal cancer** (*n* = 7196)		**Stage I and II NSCLC** (*n* = 1798)	
	**Odds ratio** **(95% CI)**	*P* value	**Odds ratio** **(95% CI)**	*P* value	**Odds ratio** **(95% CI)**	*P* value
No dementia	Reference		Reference		Reference	
Mild dementia	1.02 (0.65–1.59)	0.933	1.57 (1.12–2.21)	0.010	0.29 (0.17–0.49)	< 0.001
Moderate-to-severe dementia	0.12 (0.06–0.24)	< 0.001	0.96 (0.55–1.66)	0.882	0.19 (0.05–0.82)	0.026
	Model 5: Adjusted odds ratios of receiving pharmacotherapy					
	**Stage IV gastric cancer** (*n* = 969)				**Stage III and IV NSCLC** (*n* = 2168)	
	**Odds ratio** **(95% CI)**	*P* value			**Odds ratio** **(95% CI)**	*P* value
No dementia	Reference				Reference	
Mild dementia	0.50 (0.22–1.13)	0.095			0.38 (0.23–0.62)	< 0.001
Moderate-to-severe dementia	0.41 (0.11–1.49)	0.175			0.22 (0.08–0.56)	0.002

All odds ratios are adjusted for age, sex, comorbidities, and stage

*CI* confidence interval, *NSCLC *non-small cell lung cancer

^a^Cancer treatment includes tumour resection, pharmacotherapy, and radiotherapy

^b^Tumour resection includes endoscopic resection, open surgical resection, laparoscopic resection, and thoracoscopic resection

Supplementary Table 1 presents the association between dementia and standard cancer treatment stratified by cancer stage after adjusting for age, sex, and comorbidities. Patients with mild dementia had significantly higher odds of undergoing tumour resection for stage III colorectal cancer than those without dementia.

### Impact of dementia on mortality after cancer diagnosis

Table 4 presents the adjusted hazard ratios of all-cause mortality for mild dementia and moderate-to-severe dementia (vs. no dementia). In all three cancer sites, mild dementia and moderate-to-severe dementia were associated with an approximately two- to three-fold increase in the hazard of all-cause mortality even after adjusting for potential confounders. The effect size was greater for moderate-to-severe dementia than mild dementia, as shown in the higher point estimates of the adjusted hazard ratios. The adjusted estimates of the potential confounders showed that older age, male sex, comorbidities, advanced-stage cancer, unstaged cancer, and small cell lung cancer were associated with an increased hazard of all-cause mortality in the three cancer sites.

**Table 4:** Associations between dementia status and all-cause mortality within 3 years of cancer diagnosis in older adults

	Gastric cancer (*n* = 6016)		Colorectal cancer (*n* = 7254)		Lung cancer (*n* = 4490)	
	Hazard ratio (95% CI)	*P* value	Hazard ratio (95% CI)	*P* value	Hazard ratio (95% CI)	*P* value
Dementia						
No dementia	Reference		Reference		Reference	
Mild dementia	1.85 (1.54–2.22)	< 0.001	1.97 (1.65–2.34)	< 0.001	1.80 (1.54–2.10)	< 0.001
Moderate-to-severe dementia	3.17 (2.43–4.12)	< 0.001	3.60 (2.83–4.59)	< 0.001	2.57 (2.00–3.30)	< 0.001
Age, years						
65–69	Reference		Reference		Reference	
70–74	1.14 (0.99–1.31)	0.060	1.18 (1.01–1.38)	0.042	1.18 (1.05–1.32)	0.004
75–79	1.51 (1.31–1.73)	< 0.001	1.55 (1.33–1.80)	< 0.001	1.39 (1.24–1.56)	< 0.001
80–84	2.28 (1.97–2.63)	< 0.001	2.12 (1.80–2.49)	< 0.001	2.18 (1.92–2.46)	< 0.001
≥ 85	2.96 (2.50–3.49)	< 0.001	3.41 (2.87–4.05)	< 0.001	2.85 (2.43–3.35)	< 0.001
Sex						
Female	Reference		Reference		Reference	
Male	1.17 (1.06–1.29)	0.002	1.26 (1.14–1.39)	< 0.001	1.76 (1.61–1.92)	< 0.001
Comorbidity						
No comorbidity	Reference		Reference		Reference	
Moderate comorbidities	1.49 (1.35–1.65)	< 0.001	1.58 (1.41–1.76)	< 0.001	1.18 (1.08–1.29)	< 0.001
Severe comorbidities	1.91 (1.54–2.37)	< 0.001	2.82 (2.22–3.58)	< 0.001	1.45 (1.21–1.73)	< 0.001
Cancer stage						
0	—		0.77 (0.62–0.96)	0.021	—	
I	Reference		Reference		Reference	
II	2.55 (2.15–3.01)	< 0.001	1.24 (1.01–1.51)	0.037	2.62 (2.17–3.15)	< 0.001
III	5.37 (4.67–6.17)	< 0.001	2.16 (1.80–2.60)	< 0.001	5.29 (4.58–6.11)	< 0.001
IV	19.01 (16.95–21.31)	< 0.001	12.44 (10.50–14.75)	< 0.001	11.38 (9.98–12.97)	< 0.001
Unstaged	11.50 (8.99–14.72)	< 0.001	10.83 (7.76–15.10)	< 0.001	10.03 (7.63–13.18)	< 0.001
Histology						
Non-small cell lung cancer	—		—		Reference	
Small cell lung cancer	—		—		1.25 (1.12–1.40)	< 0.001

*CI* confidence interval

## Discussion

The primary aim of this study was to characterise the cancer pathways in older adults with and without preexisting dementia. Our analysis offers four key findings. First, older patients with dementia were more likely to be diagnosed with unstaged or advanced-stage cancer than their non-dementia counterparts. Second, patients with dementia were less likely to receive treatment for gastric and lung cancer, but more likely to receive treatment for colorectal cancer. Third, patients with dementia had a higher hazard of all-cause mortality within three years after receiving a cancer diagnosis. Fourth, the effects of moderate-to-severe dementia on cancer stage, treatment, mortality were greater than those of mild dementia, with the exception of treatment for colorectal cancer. These results corroborate the relatively scarce literature regarding older patients with preexisting dementia and cancer, although our finding that patients with mild dementia were more likely to receive treatment for colorectal cancer was inconsistent with previous studies [10–18]. Our study was strengthened by the analysis of dementia severity in a multicentre dataset with a large sample size, which allowed us to consider the exposure-response relationships between preexisting dementia and the various outcomes. Furthermore, our study covered several common cancer sites that were analysed using separate models.

The observation that older adults with dementia were more likely to be diagnosed with unstaged cancer may be attributable to a variety of factors. One possible explanation could be that these patients undergo less diagnostic activity because such procedures are often invasive and/or painful [30]. In view of the uncertain survival benefits from cancer treatment for patients with dementia, the reduced use of invasive diagnostic procedures may reflect an extension of clinicians’ clinical judgement [11, 12]. Next, we found that patients with dementia were skewed toward advanced-stage cancer at the time of diagnosis. This could be explained by delays in diagnosing these patients, which may arise from their diminished ability to recognise symptoms or to seek help during the early stages of disease [31]. Another explanation could be that such patients are unlikely to undergo routine cancer screenings, which would hinder the early detection of cancer [32]. However, caution is needed when comparing our results with those of previous studies in other countries. The proportions of patients with advanced-stage cancer within a cohort are dependent on the prevalence of cancer screening and the characteristics of the health care delivery system. Furthermore, our study population focused on hospitalised patients, which would likely have different characteristics from patients treated in outpatient settings. In our study, stage IV colorectal cancer accounted for 18.3% and 26.1% (data not shown) of staged cancer in patients with mild dementia and moderate-to-severe dementia, respectively. In contrast, the corresponding values in the US were 19.8–20.5% [10–12].

Dementia was found to be associated with a decreased likelihood of tumour resection for gastric cancer, as well as a decreased likelihood of tumour resection and pharmacotherapy for NSCLC. These results can be interpreted in several ways, such as concerns regarding additional staff time for obtaining informed consent for procedures, and the perceived limited benefits of cancer treatment for older adults with dementia [8]. Cognitive impairment can complicate the process of informed consent (e.g., patients are less able to comprehend explanations about the procedures, and difficulties in obtaining agreement from caregivers who know the patient well), thereby limiting the treatment options that clinicians are willing to recommend [11]. Furthermore, such concerns may be spurred by limited available evidence regarding the stage-specific benefits, risks, and tolerances of cancer treatment in patients with dementia. At present, clinical oncologists must work with insufficient evidence from prospective studies or oncology guidelines for the management of older patients with dementia because these individuals are often excluded from clinical trials [9, 33]. Practice guidelines based on evidence from clinical trials involving young participants or older participants without cognitive impairments may not provide relevant insight into the overall benefits for older adults with dementia [10]. It is therefore possible that the decreased likelihood of treatment for gastric cancer and NSCLC in older adults with dementia may not be indicative of undertreatment, but instead reflect reasonable clinical judgement [12, 34].

In contrast, mild dementia was associated with an increased likelihood of tumour resection for colorectal cancer. Our stage-stratified analyses revealed that this finding could be mainly attributable to patients with mild dementia who have stage III colorectal cancer. One possible explanation may be the availability of treatment alternatives (e.g., endoscopic stenting) for obstructive lesions. Guidelines published in 2014 recommend stents as the preferential treatment for the palliation of obstructive cancer [35]. For older adults believed to be poor surgical candidates by their physicians, a symptom-directed approach such as stenting may be reasonable in elective care settings, although those who avoided an elective colectomy at the time of diagnosis may eventually require delayed surgical intervention. On the other hand, patients presenting with acute colonic obstruction may need emergency surgical intervention for decompression. In the mid-2010s, immediate surgery was more common than stenting as a bridge-to-surgery for such cases [36, 37]. Furthermore, older adults with dementia are more likely to present with colorectal cancer as an emergency admission than their non-dementia counterparts [38]. These facts may have contributed to our observation that patients with dementia had an increased likelihood of tumour resection. Moreover, this finding is also supported by the rules of Japanese cancer registries, which stipulate the documentation of any emergency surgery (but not endoscopic stenting or delayed surgical procedure) performed at the time of cancer diagnosis. However, this conflicts with earlier reports that such patients tend to choose nonsurgical management for colon cancer [12, 13]. Further studies are therefore needed to characterise the treatment patterns for colorectal cancer in patients with dementia. It should also be noted that we observed no significant association between moderate-to-severe dementia and the likelihood of tumour resection for colorectal cancer. A plausible explanation is that the concurrent presence of a driving factor and an inhibiting factor for tumour resection cancelled each other out: although patients with moderate-to-severe dementia are more likely to require emergency surgery due to acute presentation, clinicians may hesitate to recommend surgical procedures for vulnerable adults with a high risk of postsurgical complications. The mutual cancellation of these opposing factors could explain our observed lack of association between moderate-to-severe dementia and tumour resection.

Older adults with dementia were found to have poorer survival after receiving a cancer diagnosis than their non-dementia counterparts. Potential explanations include a decreased likelihood of cancer treatment, an increased risk of cancer treatment-related complications, and the influence of other health problems [10, 11, 13, 15–18]. There may also be differences in cause-specific mortality between patients with and without dementia [10], and understanding how preexisting dementia affects mortality from various causes could inform oncological care decisions.

### Implications

Our findings indicate that there are numerous points of improvement for oncological care in older patients with dementia, especially in light of the projected increases in cancer patients with preexisting dementia [6]. Given the current paucity of relevant research, clinicians must make decisions with limited support from evidence-based guidelines when older adults with dementia present with cancer [9, 33]. Guidelines that outline cancer treatment strategies in patients with dementia could support clinicians in their clinical judgement of appropriate treatment. Further research is therefore needed to establish a more robust evidence base to guide treatments for patients with both conditions. Due to the lack of national and international guidelines, academic societies and hospitals may need to develop their own institutional guidelines or manuals on cancer screening and treatment for patients with dementia. Our findings on the differences in cancer pathways according to dementia status could contribute to the development of such protocols. Moreover, referrals to multidisciplinary teams consisting of geriatricians, liaison psychiatrists, and clinical nurse specialists prior to cancer treatment may help clinicians with the processing, planning, and delivery of care. This could reduce the burden on clinicians when treating patients with dementia [8].

### Limitations

This study had several limitations. First, there was no available information on the presence or absence of caregivers despite their crucial role in treatment decisions for older adults with dementia. Also, our dataset did not allow us to evaluate the decision-making process for oncological care. Second, our study population may underrepresent older adults with dementia and suspected cancer as not all these patients would be referred to or hospitalised in the accredited cancer hospitals for diagnostic investigation or cancer treatment. As a result, our findings may not be indicative of the nationwide prevalence of patients with both conditions. As our study population only included hospitalised patients, our results may underestimate the likelihood of patients with dementia to be diagnosed with unstaged cancer and overestimate the likelihood of such patients to receive treatment for cancer. Also, our study population may be overrepresented by patients with emergency admissions due to acute presentation, which could explain our findings on the increased likelihood of tumour resection for colorectal cancer in patients with mild dementia. Third, there was no available information on the causes of death, which may have offered insight into the shorter survival times observed in patients with dementia. Fourth, we did not have access to information that would distinguish cancer treatments with curative intent from those with palliative intent. In addition, cancer treatments omitted palliative procedures (e.g., endoscopic stenting) that did not reduce tumour mass. Despite these limitations, this observational study provides valuable evidence on the differences in cancer pathways between patients with and without dementia in actual clinical practice.

## Conclusion

Older adults with preexisting dementia presented with patterns of cancer staging, treatment, and all-cause mortality that were distinct from those of patients without dementia. Such patients are less likely to receive standard cancer treatment and more likely to experience poorer outcomes. Clinicians should be aware of these risks, and would benefit from standardised guidelines to aid their decision-making in diagnosing and treating these patients.

## Supplementary Information

**Additional file 1: Supplementary Table 1.** Stage-stratified associations between dementia status and standard cancer treatment in older adults.

## Data Availability

The data that support the findings of this study are available on reasonable request from the corresponding author. The data are not publicly available due to privacy and ethical restrictions.

## Abbreviations

CCI: Charlson Comorbidity Index
CI: confidence interval
ICD-O-3: International Classification of Diseases for Oncology Third Edition
IQR: interquartile range
NSCLC: non-small cell lung cancer
