# Supplementary material for: Patterns of staging, treatment, and mortality in gastric, colorectal, and lung cancer among older adults with and without preexisting dementia: a Japanese multicentre cohort study
Source: BMC Cancer. 2023 Jan 19;23:67. doi: 10.1186/s12885-022-10411-y (PMC9854163; doi:10.1186/s12885-022-10411-y)
Supplement: Supplementary file 1 — Additional file 1: Supplementary Table 1. Stage-stratified associations between dementia status and standard cancer treatment in older adults. [file 12885_2022_10411_MOESM1_ESM.pdf]

Supplementary Table 1. Stage-stratified associations between dementia status and standard cancer treatment in older adults.

	Odds ratio (95% CI)	*P* value	Odds ratio (95% CI)	*P* value	Odds ratio (95% CI)	*P* value
			Tumour resection[a] for stage 0 CRC (n=1916)			
No dementia			Reference			
Mild dementia			0.99 (0.12–8.07)	0.994		
Moderate-to-severe dementia			0.05 (0.01–0.28)	<0.001		
	Tumour resection for stage I GC (n=3756)		Tumour resection for stage I CRC (n=1495)		Tumour resection for stage I NSCLC (n=1424)	
No dementia	Reference		Reference		Reference	
Mild dementia	0.59 (0.34–1.04)	0.068	0.88 (0.40–1.94)	0.760	0.30 (0.17–0.53)	<0.001
Moderate-to-severe dementia	0.11 (0.05–0.22)	<0.001	0.22 (0.06–0.81)	0.023	0.25 (0.05–1.17)	0.079
	Tumour resection for stage II GC (n=573)		Tumour resection for stage II CRC (n=1431)		Tumour resection for stage II NSCLC (n=374)	
No dementia	Reference		Reference		Reference	
Mild dementia	1.15 (0.50–2.67)	0.738	1.25 (0.55–2.84)	0.594	0.31 (0.08–1.23)	0.096
Moderate-to-severe dementia	0.20 (0.03–1.25)	0.085	0.65 (0.24–1.77)	0.401	0.00 (0.00–>1000)	>0.99
	Tumour resection for stage III GC (n=622)		Tumour resection for stage III CRC (n=1430)		Pharmacotherapy for stage III NSCLC (n=717)	
No dementia	Reference		Reference		Reference	
Mild dementia	1.80 (0.80–4.06)	0.154	2.47 (1.32–4.63)	0.005	0.36 (0.08–1.58)	0.176
Moderate-to-severe dementia	0.79 (0.19–3.31)	0.752	2.63 (0.79–8.74)	0.114	0.00 (0.00–>1000)	>0.99
	Pharmacotherapy for stage IV GC (n=969)		Tumour resection for stage IV CRC (n=924)		Pharmacotherapy for stage IV NSCLC (n=1451)	
No dementia	Reference		Reference		Reference	
Mild dementia	0.50 (0.22–1.13)	0.095	1.54 (0.85–2.77)	0.153	0.38 (0.23–0.64)	<0.001
Moderate-to-severe dementia	0.41 (0.11–1.49)	0.175	1.35 (0.57–3.20)	0.500	0.25 (0.09–0.66)	0.005

CI, confidence interval; CRC, colorectal cancer; GC, gastric cancer; NSCLC, non-small cell lung cancer. All odds ratios are adjusted for age, sex, and comorbidities.

[a]Tumour resection includes endoscopic resection, open surgical resection, laparoscopic resection, and thoracoscopic resection.
